# Granulomatosis With Polyangiitis: Cardiac, Renal, and Respiratory Involvement

**DOI:** 10.7759/cureus.61529

**Published:** 2024-06-02

**Authors:** Pooja Khatiwala, Parita Patel, Alexandria Nachodsky

**Affiliations:** 1 Internal Medicine, Cooper University Hospital, Camden, USA

**Keywords:** acute renal failure and hemodialysis in icu, submassive pulmonary embolism, diffuse alveolar hemorrhage, small vessel vasculitis, granulomatosis with polyangiitis (gpa)

## Abstract

Granulomatosis with polyangiitis (GPA), formerly known as Wegener’s granulomatosis, is an anti-neutrophilic cytoplasmic autoantibody (ANCA)-associated small-vessel vasculitis. Typically, it causes upper and lower respiratory tract necrotizing granulomatous inflammation and necrotizing glomerulonephritis. The diagnosis is made through clinical symptoms, positive antibody testing, imaging, and kidney biopsy. We describe the case of a man in his 60s who presented with multiple complications of GPA including rapidly progressive renal failure requiring dialysis, diffuse alveolar hemorrhage, acute respiratory distress syndrome (ARDS), circulatory shock, submassive pulmonary embolism, and biventricular and dilated cardiomyopathy.

## Introduction

Granulomatosis with polyangiitis (GPA) affects men and women equally and the onset of disease occurs around 65-74 years of age. The incidence is 13 cases per million person-years. Although the exact cause is unknown, there are environmental and genetic factors involved. Major histocompatibility complex II (MHC II) genes are associated with an increased risk of developing GPA. *HLA*-DPB1*04 has been identified as a risk allele. Some non-MHC genes, such as *PRTN3*, *SERPINA1*, *SEMA6A*, and *CTLA-4*, have also been associated with an increased risk of GPA. There are infectious agents such as bacterial, fungal, and viral infections of the ears, nose, and respiratory tract that can trigger GPA. Certain drugs such as antithyroid medications and anti-hypertensives have also been associated with increased risk [1].

Rapidly progressive glomerulonephritis is a serious manifestation of GPA [2]. Patients with GPA have an increased risk of venous thromboembolism (VTE), which includes deep vein thrombosis (DVT) and pulmonary embolism (PE), especially during times of active GPA disease [3]. Cardiac involvement is a rare complication of GPA with an estimated incidence of 3.3% [4]. Respiratory involvement is common due to the necrotizing granulomatous inflammation that is produced in the upper and lower airways [1]. This case is an important addition to the literature due to the serious renal, cardiac, and respiratory complications presented in this patient, such as end-stage renal disease (ESRD), dilated cardiomyopathy, and diffuse alveolar hemorrhage. 

## Case presentation

A male in his 60s initially presented with dyspnea and atrial fibrillation with rapid ventricular response (RVR) and was thereafter found to have new onset severe heart failure with reduced ejection fraction (HFrEF) and multiple other complications of GPA. In his initial presentation, he had severe left ventricular (LV) dysfunction with an ejection fraction of 12% with severe global hypokinesis. He underwent left heart catheterization, which demonstrated nonobstructive coronary artery disease. He underwent direct current cardioversion (DCCV) and was placed on amiodarone and apixaban upon discharge. The patient was recommended to follow up in the outpatient department for an MRI to rule out causes for cardiomyopathy.

The patient presented approximately a year later with progressive lethargy, lower extremity, and pelvic pain and was noted to have acute anemia and acute renal failure. He was also noted to be tachycardic and mildly hypotensive with a blood pressure of 92/67 mmHg. Hemoglobin was 8.8 g/dl (it was 14.3 g/dl at the initial presentation) and creatinine level of 3.4 mg/dl. 

**Figure 1:**
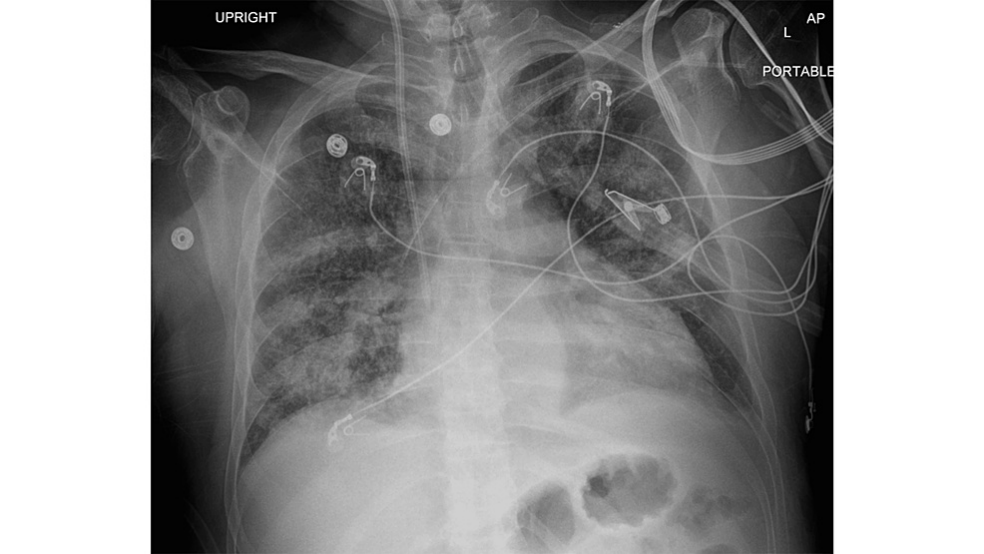
Chest X-ray

On admission, the patient had a urinalysis done that was significant for granular casts, large amounts of red blood cells (RBCs), and elevated protein. It was revealed that he also had a positive cytoplasmic anti-neutrophilic cytoplasmic autoantibody (c-ANCA) with a titer level of 1:320 and a positive proteinase 3 (PR3) level of 23.7 antibody index, which was diagnostic of GPA. The patient was found to be negative for the anti-glomerular basement membrane (anti-GBM) antibody and the myeloperoxidase (MPO) antibody. He also had a chest x-ray (CXR) done, which was concerning for multifocal pneumonia. The CXR showed perihilar predominant airspace opacities that were most likely edema with no evidence of pneumothorax (Figure 1). 

There was concern for possible alveolar hemorrhage due to the vasculitis so a bronchoalveolar lavage (BAL) was completed. There were alveolar macrophages that were positive with iron staining, benign squamous cells, and no malignant cells were seen. The patient was then diagnosed with acute hypoxic respiratory failure and acute respiratory distress syndrome, which required intubation and caused shock. This was detected through arterial blood gas (ABG), CXR, and clinical symptoms. The patient's ABG showed a pH of 7.17 with a partial pressure of arterial carbon dioxide (PaCO2) of 62, partial pressure of oxygen (PaO2) of 89, and bicarbonate (HCO3) of 19 while on room air.

**Figure 2:**
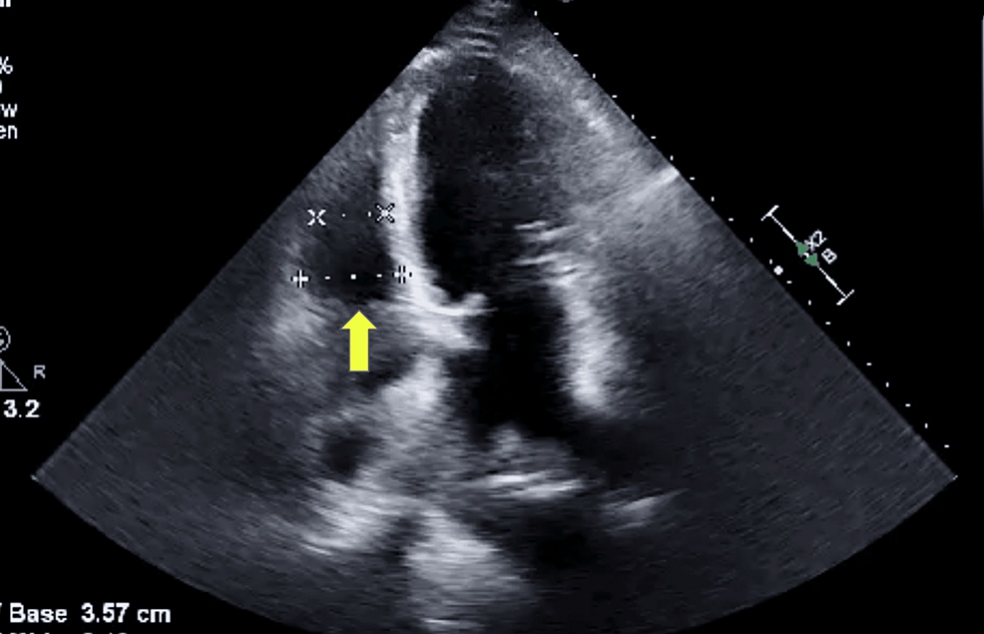
Echocardiography showing moderately dilated right ventricle (yellow arrow)

He was then placed in the ICU due to the concern for shock that required pressor support. Due to his progressive hypoxic respiratory failure, he was quickly intubated and sedated to maintain ventilator synchrony. The patient had echocardiography done that showed a mildly dilated left ventricle with normal wall thickness. His systolic function was severely impaired. The LVEF was estimated at 20-25%. There was also evidence of a moderately dilated right ventricle with severely reduced function (Figure 2).

Due to the diffuse alveolar hemorrhage and newly diagnosed vasculitis, the patient was also started on plasma exchange (PLEX). He received a total of seven days of PLEX. His volume was being exchanged with fresh frozen plasma (FFP). Due to the concern for pneumonia on imaging, the patient was also given antibiotics such as ceftriaxone and doxycycline. He was seen by nephrology who recommended IV iron sucrose for his acute anemia and continued monitoring of his renal function. The patient’s creatinine continued to increase up to 6.4 mg/dl so he was re-evaluated by nephrology. He was then started on continuous renal replacement therapy (CCRT). The patient also received rituximab, cyclophosphamide, and methylprednisolone for vasculitis. The patient refused further rituximab treatment after one dose due to lower extremity cramping and nausea following transfusion. Due to the patient’s refusal, Rheumatology recommended IV cyclophosphamide 10 mg/kg induction for treatment, which was repeated after 14 days of initial infusion. After three days of methylprednisolone, he was transitioned to prednisone 40 mg. Due to the concern for *Pneumocystis jiroveci* pneumonia (PJP) acquisition from the immunosuppressive effects of cyclophosphamide, the patient was started on trimethoprim-sulfamethoxazole (TMP-SMX). Eventually, his respiratory status started to improve, and he was extubated. 

The patient had a complicated hospital course and was observed in the hospital for two weeks before discharge. He was transitioned to intermittent hemodialysis from CRRT. He was discharged with 40 mg of prednisone for treatment of his GPA. After discharge, the patient was sent to a sub-acute rehabilitation facility and was established at a hemodialysis center for outpatient treatment. 

Within one month of the patient being discharged, he was readmitted to the hospital with shortness of breath and pleuritic chest pain found to be in atrial fibrillation with RVR, submassive pulmonary emboli, acute hypoxic respiratory failure requiring supplemental nasal cannula oxygen, borderline low blood pressure and diffuse interstitial pulmonary edema. He presented with pleuritic chest pain and shortness of breath. On CT, he was found to have bilateral pulmonary emboli and diffuse interstitial pulmonary edema. The patient required an upgrade to the ICU and subsequently underwent a thrombectomy. The patient has a history of atrial fibrillation but was not prescribed an anticoagulant before his admission due to his history of anemia, cardioversion, and recent alveolar hemorrhage. He was discharged to a rehabilitation facility with apixaban. The patient thereafter had another admission a few weeks later for chest pain and palpitations caused by atrial fibrillation with RVR, in which his medications were further adjusted by the cardiology team. The patient is now discharged to a subacute rehabilitation facility. 

## Discussion

Granulomatosis with polyangiitis is a systemic, small-vessel, noncaseating granulomatous vasculitis that typically presents in men around 50 years of age. Patients can present with pulmonary symptoms, such as cough, hemoptysis, and sinus symptoms. They can also present with renal dysfunction issues such as proteinuria and hematuria. The lungs and kidneys are involved in 95% and 80% of cases, respectively, and less commonly, the skin (50%) and the eyes (45%) [5]. The pathogenesis is an activation of the immune system to produce the c-ANCA antibody, which causes widespread necrotizing and granulomatous inflammation. The multisystem involvement and the varied symptoms of GPA make diagnosis and treatment difficult. For better prognosis for patients, early diagnosis and treatment are vital [6]. 

Our patient presented with renal symptoms, which are very severe and the leading cause of mortality in GPA [2]. He had significant proteinuria and hematuria, which is suggestive of GPA-associated glomerulonephritis. Glomerulonephritis is the most common renal pathology affecting 70-85% of people with GPA but only 10-15% of patients present with severe renal inadequacy with creatinine over 2 mg/dL [6]. Though over 70% of patients can present with renal symptoms, usually this takes over two years to develop [7]. Our patient’s disease rapidly progressed, and he had a need for dialysis within days of diagnosis. The disease process of renal involvement can be indolent or progress to ESRD in days [6]. Our patient had this rapid progression to ESRD. Renal involvement has been shown to be a determinant of poor prognosis in patients with GPA [8]. Patients with renal involvement that have functional impairment of the kidneys characterized by high creatinine and low glomerular filtration rate (GFR) have a higher risk of death. They also have a significant increase in the mean length of hospital stay compared to patients without renal involvement (8.14 days vs 6.59 days). Readmissions for patients with GPA also increase the mean length of stay in the hospital (8.0 vs 7.2 days). This indicates a higher level of complexity in patients who have GPA with renal complications and a greater burden to the healthcare system [8]. In the case of our patient with many social barriers to continuous care, his consistent need for hemodialysis and follow-up with Nephrology proved difficult and was the reason for his constant readmissions. 

GPA is also the most common pulmonary vasculitis. It affects the lung parenchyma and causes symptoms such as cough, dyspnea, and diffuse alveolar hemorrhage [6]. The mainstay of treatment for GPA is cyclophosphamide started at the same time as methylprednisolone, followed by a high-dose steroid [2]. PLEX is also used for patients with GPA who present with rapidly progressive glomerulonephritis and pulmonary hemorrhage. Patients with severe respiratory failure can benefit greatly from PLEX, as they require less time in the ICU [9]. It has also been shown that TMP-SMX can be given to patients with GPA to keep them in remission from their symptoms [2]. Our patient was given all the above treatments to help with his course. For patients with GPA, CXR can often show pulmonary infiltrates, nodules, or cavitations, which can aid in diagnosis [1].

GPA has a very high incidence of venous thrombosis. The incidence is especially high in the active phase of the disease right after diagnosis [10]. The incidence rate of venous thrombosis in a patient with GPA is much higher compared to the average population and to patients with lupus or rheumatoid arthritis [3]. It is unknown why people with GPA have a higher incidence of venous thrombosis. However, it is suspected that they are in a more coagulable state with higher levels of factor VIII, which promotes endothelial activation and dysfunction [10]. Our patient was also not on anticoagulation for his atrial fibrillation for some time due to recent alveolar hemorrhage and anemia, which again puts him at higher risk in addition to the above.

Our patient's initial presentation was atrial fibrillation with RVR with non-ischemic cardiomyopathy. Cardiac involvement in GPA is rare and the mechanism of how this occurs is not known. It is thought that the presence of necrotizing vasculitis in small vessels contributes to cardiac involvement. Pericarditis, myocarditis, and conduction defects are the most common complications. Dilated cardiomyopathy with congestive heart failure (CHF) is exceedingly rare as a cardiac manifestation of GPA and has only been reported in eight cases since 1936 [11-17].

The patient's left heart catheterization demonstrated normal coronary arteries, and he did not have any significant medical history including hypertension and otherwise no prodromal symptoms to suggest viral etiology for cardiomyopathy. He also did not have significant alcohol use or a history of medical conditions such as HIV that can cause cardiomyopathy. Cardiac MRI along with measuring erythrocyte sedimentation rate (ESR) and C-reactive protein (CRP) can be useful in diagnosis, but endomyocardial biopsy is still the gold standard [4]. It remains unclear whether the disease process itself or a different etiology such as undiagnosed prolonged atrial fibrillation could have caused this presentation. It is possible that GPA caused myocardial injury many months before the rapid progression of the disease; however, without the MRI or biopsy, this remains unclear. Patients who present with symptoms of acute CHF and worsening renal function should have GPA included in their differential diagnoses [4].

## Conclusions

Granulomatosis with polyangiitis can be mistaken for other conditions and have a delayed treatment course. When patients present with injuries to multiple systems, such as cardiac, respiratory, and renal, early immunological testing can be very important. Early diagnosis and treatment are crucial for improved mortality in patients with GPA. Cardiac MRI and diagnostic serologic tests for GPA can allow for prompt diagnosis and management of complications from systolic dysfunction caused by dilated cardiomyopathy. Treatment with steroids and biologics can improve outcomes for patients. Multidisciplinary care is also very important in patients with GPA for recovery and maintenance of remission.
